# C-954-03. Burn Care and the Safety-net: A Cross-sectional Analysis of U.S. Acute Care Hospitals

**DOI:** 10.1093/jbcr/irag033.135

**Published:** 2026-04-06

**Authors:** Jacqueline N Byrd

**Affiliations:** University of Chicago

## Abstract

**Introduction:**

Burn care is often identified as an essential health care service, but none of the three most common safety-net hospital definitions include burn care capacity. The objectives of this study were to assess how well application of current safety-net hospital definitions based on financial data capture hospitals providing burn intensive care and compare the financial viability of burn hospitals across these definitions.

**Methods:**

Burn intensive care hospitals were identified as those reporting burn intensive care beds to the Centers for Medicare and Medicaid (CMS) Healthcare Cost Report Information System (HCRIS) annual report and American Hospital Association survey. These financial reports were further analyzed for the financial health and funding sources of each hospital. To identify safety-net hospitals, we calculated the top quartile of Disproportionate Share Hospital (DSH) funding, top quartile of Medicaid inpatient days and top quartile of uncompensated care burden among all non-Critical Access Hospitals. Financial data were winsorized at the 1st and 99th percentiles.

**Results:**

95 hospitals reported having burn intensive care beds (range 2-70) and were identified as burn hospitals. 77% of these hospitals were trauma centers (level 1-4); ^2^56% were level 1 trauma centers. 12 burn hospitals were captured as a safety-net hospital under all three of the most common SNH definitions (Fig. 1), representing a total of 135 BICU beds and $83.5 million in inpatient BICU costs. 67 burn hospitals were identified as safety net hospitals across the three definitions; ^3^with 60 hospitals identified by the top quartile of DSH payments. The mean operating margin for burn hospitals defined as safety-net hospitals by any definition was -1.4% (Table 1).

**Conclusions:**

The current definitions of safety-net hospitals used in research and funding decisions do not consistently capture burn hospitals. Using DSH funding to define safety-net hospitals identified 60 burn hospitals (of 67 meeting any of the three definitions). This is notable as DSH funding reflects state-level funding decisions that may reflect recognition of the essential nature of burn care. However, these decisions can vary across states and DSH payments alone may not prevent burn unit or hospital closure.

**Applicability of Research to Practice:**

As hospitals providing essential services face federal funding and insurance reimbursement uncertainty, it is important that burn surgeons advocate for the need for access to burn care. Reliance on any one of these definitions for future funding decisions at the state and federal could threaten continued access to timely high-quality access to burn care.

**Funding for the Study:**

N/A.

